# MALAT1/miR-101-3p/MCL1 axis mediates cisplatin resistance in lung cancer

**DOI:** 10.18632/oncotarget.23483

**Published:** 2017-12-14

**Authors:** Huaqi Wang, Li Wang, Guojun Zhang, Chunya Lu, Heying Chu, Rui Yang, Guoqiang Zhao

**Affiliations:** ^1^ Department of Respiratory Medicine, The First Affiliated Hospital of Zhengzhou University, Zhengzhou, Henan 450052, P.R. China; ^2^ School of Basic Medical Sciences, Zhengzhou University, Zhengzhou, Henan, 450001, P.R. China

**Keywords:** MALAT1, cisplatin, resistance, miR-101-3p, myeloid cell leukemia 1

## Abstract

In this study, we investigated the mechanism by which lncRNA metastasis-associated lung adenocarcinoma transcript 1 (MALAT1) mediates cisplatin resistance in lung cancer. Lung cancer patients with high MALAT1 levels were associated with cisplatin resistance and low overall survival. Moreover, cisplatin-resistant A549/DDP cells showed higher MALAT1 expression than cisplatin-sensitive lung cancer cells (A549, H460, H1299 and SPC-A1). Dual luciferase reporter and RNA immunoprecipitation assays showed direct binding of miR-101-3p to MALAT1. MALAT1 knockdown in lung cancer cells resulted in miR-101-3p upregulation and increased cisplatin sensitivity. In addition, miR-101-3p decreased myeloid cell leukemia 1 (*MCL1*) expression by binding to the 3’-untranslated region (3’-UTR) of its mRNA. These results demonstrate that MALAT1/miR-101-3p/MCL1 signaling underlies cisplatin resistance in lung cancer.

## INTRODUCTION

Lung cancer is the most commonly diagnosed cancer and the leading cause of cancer-related deaths worldwide [1]. Although cisplatin-based chemotherapy is the first line treatment for lung cancer patients, the 5-year survival rates are poor because of drug resistance [2]. Therefore, identifying the underlying molecular mechanisms of drug resistance is critical for developing novel therapeutic strategies to improve treatment outcomes in lung cancer patients.

Long non-coding RNAs (lncRNA) are non-protein coding RNAs that are ≥ 200 nucleotides long [3]. They affect gene expression by regulating chromatin modification, transcription and post-transcriptional mechanisms [4]. Several lncRNAs are oncogenes that modulate survival, proliferation, metastasis and chemo-resistance of cancer cells. LncRNA FAL1 promotes tumor cell proliferation by recruiting chromatin repressor BMI1 to repress p21 [5]. LncRNA HOTAIR promotes breast cancer metastasis by recruiting Polycomb repressive complex 2 (PRC2) to alter the overall genomic landscape [6]. Moreover, HOTAIR expression correlates with differential response of ovarian cancer cells to platinum-based chemotherapy [7]. LncRNA metastasis-associated lung adenocarcinoma transcript 1 (MALAT1) was first identified as a negative prognostic biomarker in early non-small cell lung cancer [8]. It is dispensable for mouse development [9] and interacts with polycomb 2 protein to control relocation of growth-control genes between polycomb bodies and inter-chromatin granules [10]. Recently, MALAT1 has been implicated in metastasis and invasion of liver, bladder, breast and cervical cancers [11–14]. It also promotes chemo-resistance in glioblastoma, liver and prostate cancers [15–17]. High levels of MALAT1 are reported in cisplatin-resistant H460R lung cancer cells [18]. However, the mechanistic role of MALAT1 in cisplatin-resistance of lung cancer remains poorly understood. Therefore, in this study, we investigated the mechanism by which MALAT1 regulates cisplatin resistance in lung cancer.

## RESULTS

### Cisplatin-resistant lung cancer tissues and cell lines show higher MALAT1 levels

**Figure 1:**
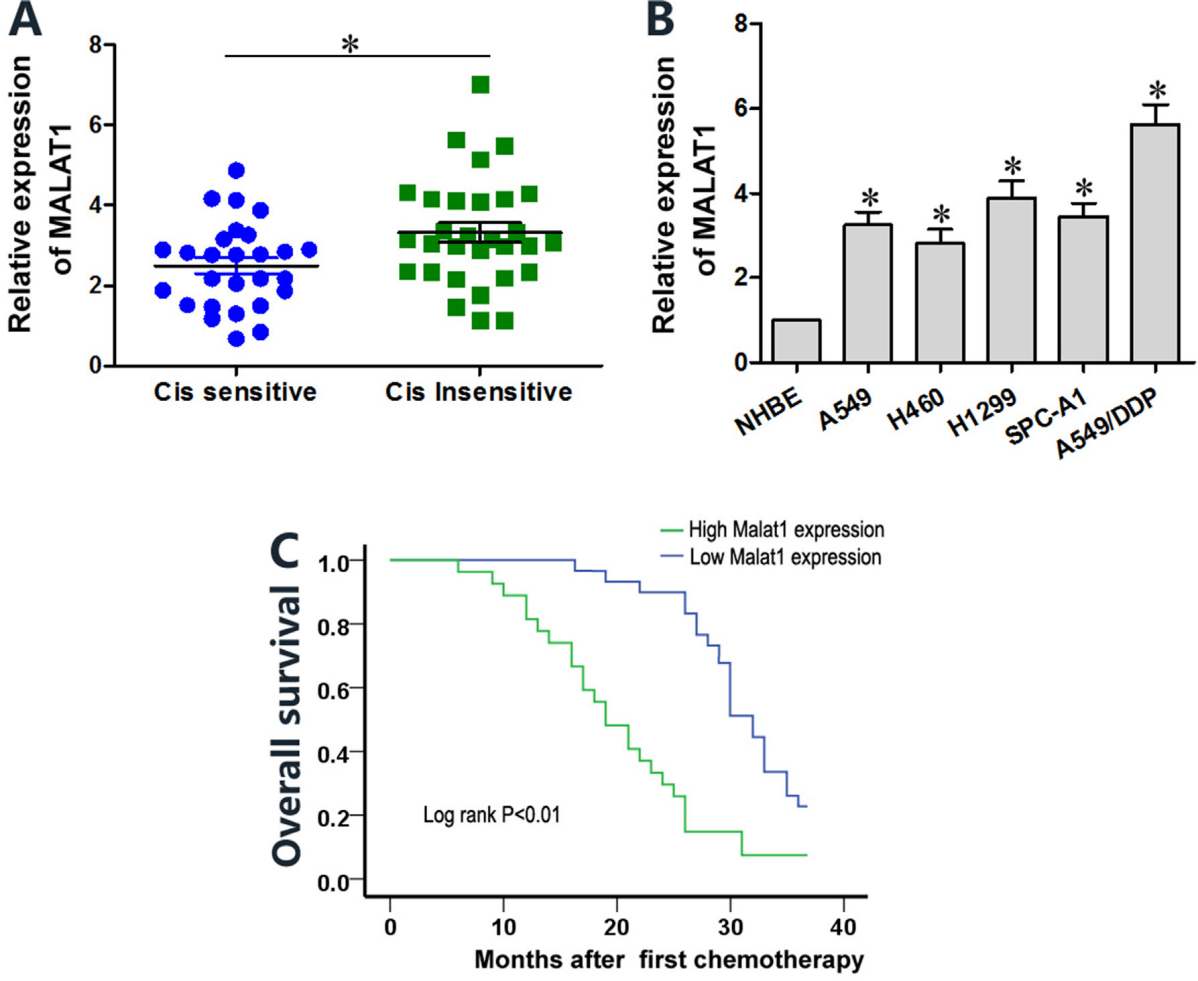
Lung cancer tissues and cell lines show high MALAT1 levels that influence cisplatin sensitivity (**A**) QRT-PCR analysis of MALAT1 levels in cisplatin-sensitive and –resistant lung cancer tissue samples (*n* = 28 per group). (**B**) QRT-PCR analysis of MALAT1 levels in normal human bronchial epithelial (NHBE) cells and A549, H1299, H469, SPC-A1 and A549/DDP lung cancer cell lines. Error bars represent mean ± S.D. from triplicate experiments. (**C**) Kaplan-Meier survival curves show overall survival of lung cancer patients with high or low MALAT1 levels. Patients with high MALAT1 expression show decreased survival than those with low MALAT1 expression (*P* < 0.01).

We analyzed MALAT1 levels in cisplatin-sensitive and -resistant lung cancer tissue samples (*n* = 28) by qRT-PCR. The overall expression of MALAT1 was higher in cisplatin-resistant lung cancer tissues than in cisplatin-sensitive samples (Figure 1A). MALAT1 levels were higher in lung cancer cell lines (A549, H460, H1299, SPC-A1 and A549/DDP) than in normal human bronchial epithelium (NHBE) cell line (Figure 1B). Moreover, MALAT1 levels were higher in the cisplatin-resistant A549/DDP cell line than in cisplatin-sensitive A549, H460, H1299 and SPC-A1 lung cancer cell lines (Figure 1B). Lung cancer patients with high MALAT1 expression demonstrated poor prognosis and low overall survival (Figure 1C). These data demonstrated that high MALAT1 expression correlates with cisplatin resistance in lung cancer.

### MALAT1 knockdown sensitizes lung cancer cells to cisplatin

**Figure 2:**
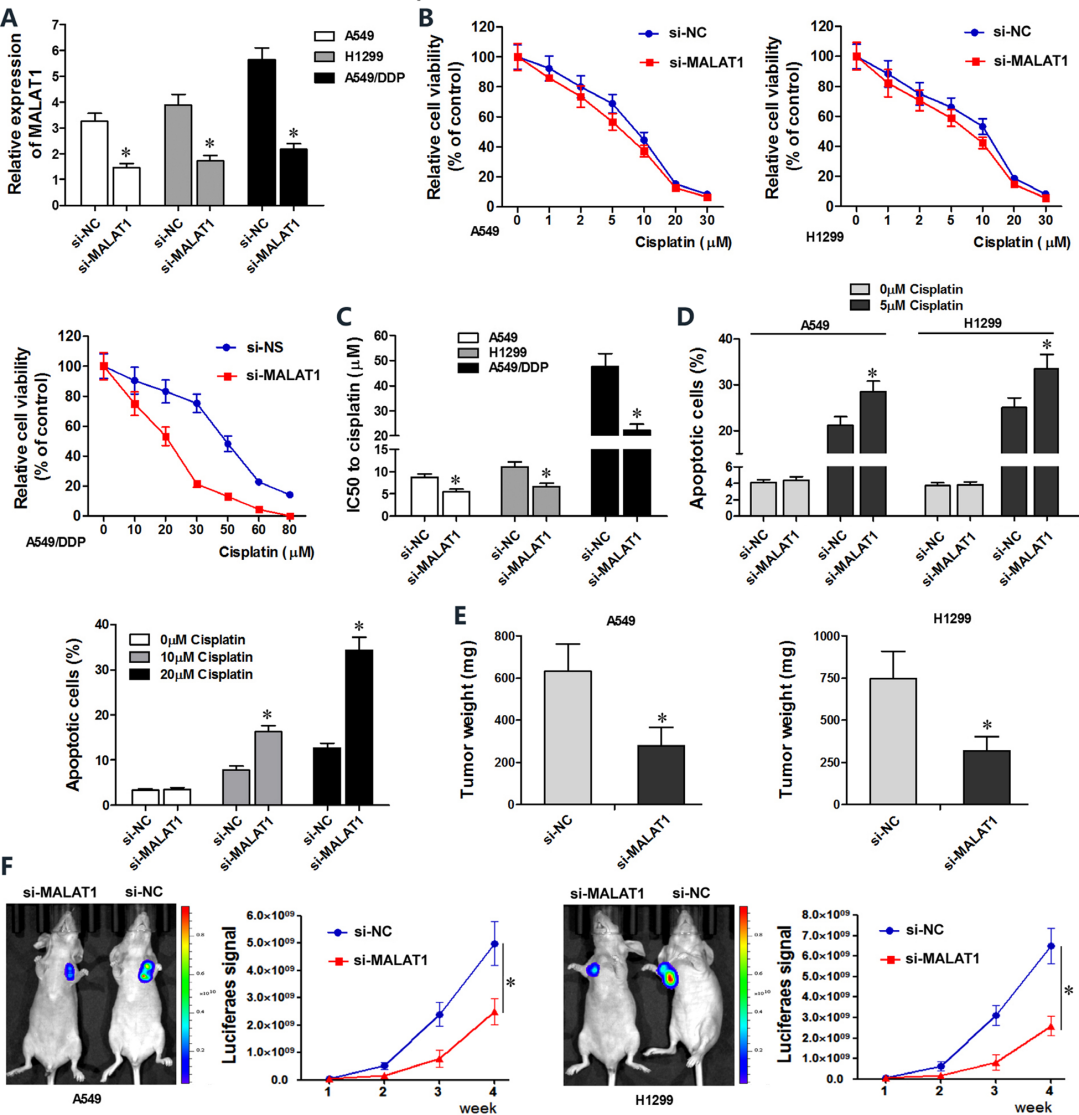
MALAT1 knockdown sensitizes lung cancer cells to cisplatin (**A**) QRT-PCR analysis of MALAT1 levels in siMALAT1- and siNC-transfected A549, H1299 and A549/DDP cells at 48 h post-transfection. (**B**) CCK-8 assay shows viability of siMALAT1- and siNC-transfected A549, H1299 and A549/DDP cells exposed to various concentrations of cisplatin (0, 1, 2, 5, 10, 20, 30 μM cisplatin for A549 and H1299 cells, and 0, 20, 30, 50, 60, 80 μM cisplatin for A549/DDP cells), 48 h post-transfection. (**C**) IC_50_ values of siMALAT1 and siNC-transfected A549, H1299 and A549/DDP cells were determined from the viability versus cisplatin concentration curves. (**D**) Flow cytometry analysis shows percent apoptosis (AnnexinV^+^ PI^+^ cells) in siMALAT1 and siNC-transfected A549, H1299 and A549/DDP cells treated with different concentrations of cisplatin (0, 5 μM cisplatin for A549 and H1299 cells, and 0, 10, 20 μM cisplatin for A549/DDP cells). (**E**) Mean weights of xenograft tumors at 4 weeks Balb/c nude mice subcutaneously injected with siMALAT1 and siNC-transfected A549 and H1299 (*n* = 5/group). (**F**) Mean size of xenograft tumors derived from siMALAT1 and siNC-transfected A549 and H1299 cells that were subcutaneously injected into Balb/c nude mice (*n* = 5/group). Bioluminescence imaging was performed in live nude mice for 4 weeks (once every week) to assess tumor growth. The intensity of chemiluminescent signal represents the size of xenograft tumors. Error bars represent mean ± S.D. from triplicate experiments.

Next, we investigated the effects of MALAT1 knockdown on chemo-sensitivity of lung cancer cells. A549, H1299 and A549/DDP cells transfected with MALAT1 siRNA (si-MALAT1) showed lower MALAT1 levels than controls (Figure 2A). CCK8 viability assay showed that MALAT1 knockdown in A549 and H1299 cells resulted in decreased viability upon cisplatin treatment and lower IC_50_ values for cisplatin (Figure 2B–2C). MALAT1 knockdown also reduced viability of cisplatin-resistant A549/DDP cells (Figure 2B–2C).

Next, we investigated if MALAT1 regulated apoptosis of lung cancer cells. Flow cytometry analysis showed that MALAT1 knockdown increased cisplatin-induced apoptosis in A549, A549/DDP and H1299 lung cancer cell lines than in corresponding controls (Figure 2D).

We performed xenograft studies in nude mice to determine the effects of MALAT1 on *in vivo* tumorigenesis. We subcutaneously injected si-MALAT1 or si-NC transfected A549 and H1299 cells that stably expressed luciferase into nude mice and assessed xenograft tumor growth by bioluminescence imaging. After 4 weeks of cisplatin exposure, the size and weight of xenograft tumors derived from siMALAT1-transfected A549 and H1299 cells were smaller than in mice xenografted with siNC-transfected A549 and H1299 cells (Figure 2E–2F). Collectively, both *in vitro* and *in vivo* experiments demonstrated that MALAT1 knockdown promotes cisplatin-induced apoptosis in lung cancer cells.

### MiR-101-3p is a direct target of MALAT1

**Figure 3:**
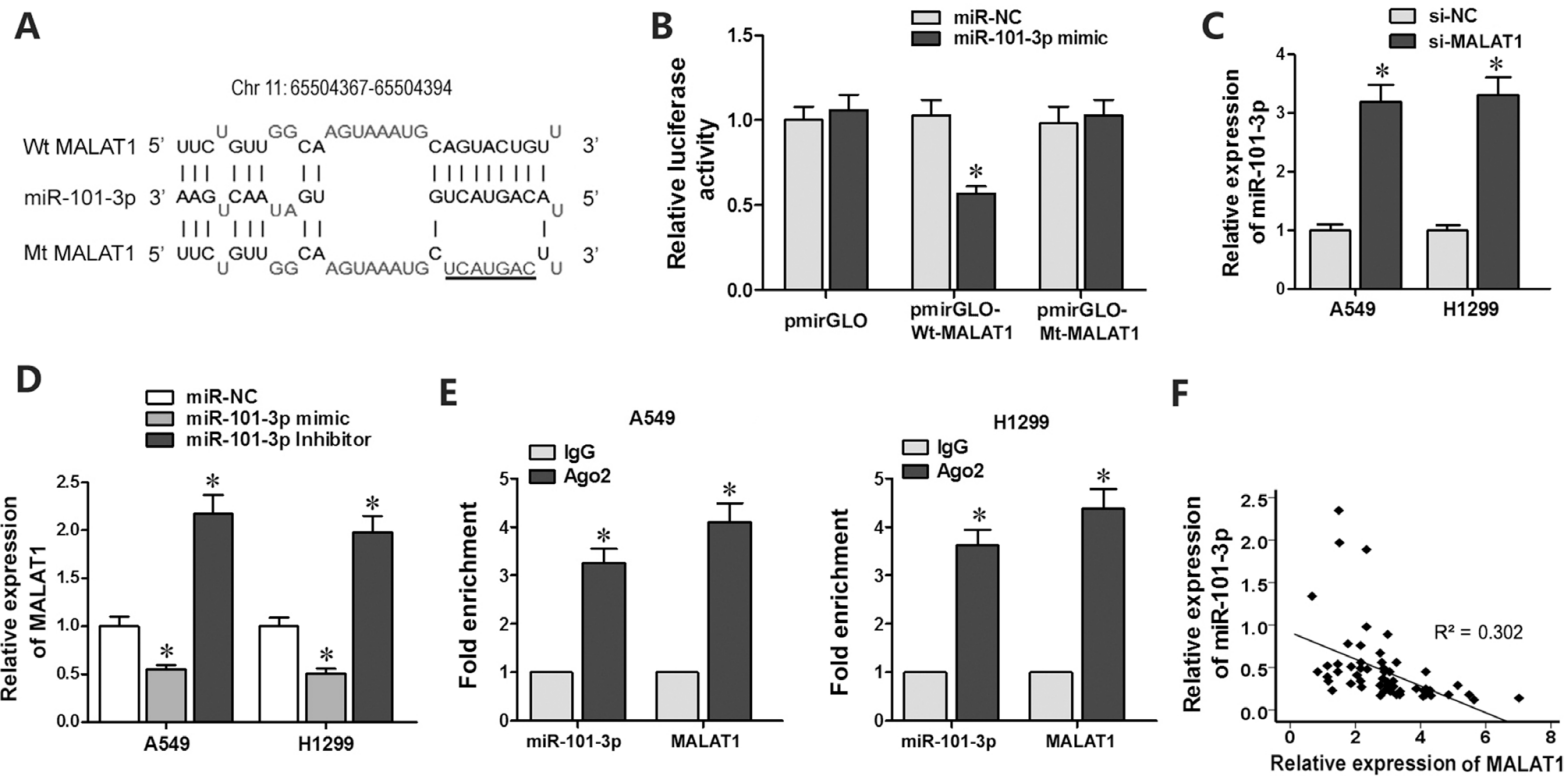
MiR-101-3p is a direct target of MALAT1 (**A**) Predicted miR-101-3p binding site in MALAT1 sequence based on DIANA-LncBase analysis. (**B**) Relative luciferase activity is decreased in cells transfected with Wt-MALAT1 (wild type miR-101-3p binding site) and miR-101-3p mimics than in cells transfected with Mt-MALAT1 (mutant miR-101-3p binding site) and miR-101-3p mimics. This demonstrates that miR-101-3p directly binds to MALAT1. (**C**) QRT-PCR analysis of miR-101-3p levels in siMALAT1 and siNC-transfected A549 and H1299 cells at 48 h post-transfection. U6 was used as internal control. (**D**) QRT-PCR analysis of MALAT1 levels in A549 and H1299 cells transfected with miR-101-3p mimics, miR-101-3p inhibitor or negative control miRNA sequence at 48h post-transfection. (**E**) QRT-PCR analysis of miR-101-3p and MALAT1 levels isolated from Ago2 and IgG immunoprecipitates derived from A549 and H1299 cells. (**F**) Pearson’s analysis shows correlation between MALAT1 and miR-101-3p levels in tissue samples from lung cancer patients sensitive or resistant to cisplatin (*n* = 28/group; Pearson’s coefficient [R] = - 0.549).

Since lncRNAs function as decoys of microRNAs, we analyzed miRNA recognition sequences in MALAT1 with the DIANA-LncBase software (http://diana.imis.athena-innovation.gr) and found a putative miR-101-3p binding site (Figure 3A). To investigate if miR-101-3p was a functional target of MALAT1, we cloned MALAT1 with the wild type (Wt-MALAT1) and mutant (Mt-MALAT1) miR-101-3p binding sites into pmirGLO-reporter plasmid. Dual luciferase reporter assay showed decreased luciferase activity in cells co-transfected with miR-101-3p mimics and pmirGLO-Wt-MALAT1 than in cells co-transfected with miR-101-3p mimics and pmirGLO-Mt-MALAT1 or miR-101-3p mimics and pmirGLO-NC (Figure 3B). This demonstrated that miR-101-3p directly binds to MALAT1.

Next, we investigated the correlation between MALAT1 and miR-101-3p expression. As showed in Figure 3C, MALAT1 knockdown increased miR-101-3p expression in A549 and H1299 cells. Moreover, transfection of miR-101-3p mimics decreased MALAT1 expression, whereas transfection of miR-101-3p inhibitor increased MALAT1 expression in A549 and H1299 cells (Figure 3D).

Ago2 protein, as a core component of RNA-induced silencing complex (RISC), binds both miRNAs and their corresponding complementary RNA molecules. We performed RNA immunoprecipitation (RIP) assay to validate direct binding between MALAT1 and miR-101-3p. QRT-PCR analysis showed that both miR-101-3p and MALAT1 were enriched in Ago2 immunoprecipitates than in control IgG immunoprecipitates from A549 and H1299 cells (Figure 3E). Furthermore, qRT-PCR analysis of lung cancer patient tissues showed that MALAT1 expression negatively correlated with miR-101-3p levels (R = -0.549, Figure 3F). These results confirmed that miR-101-3p is direct target of LncRNA MALAT1.

### MiR-101-3p upregulation enhances cisplatin sensitivity of lung cancer cells

**Figure 4:**
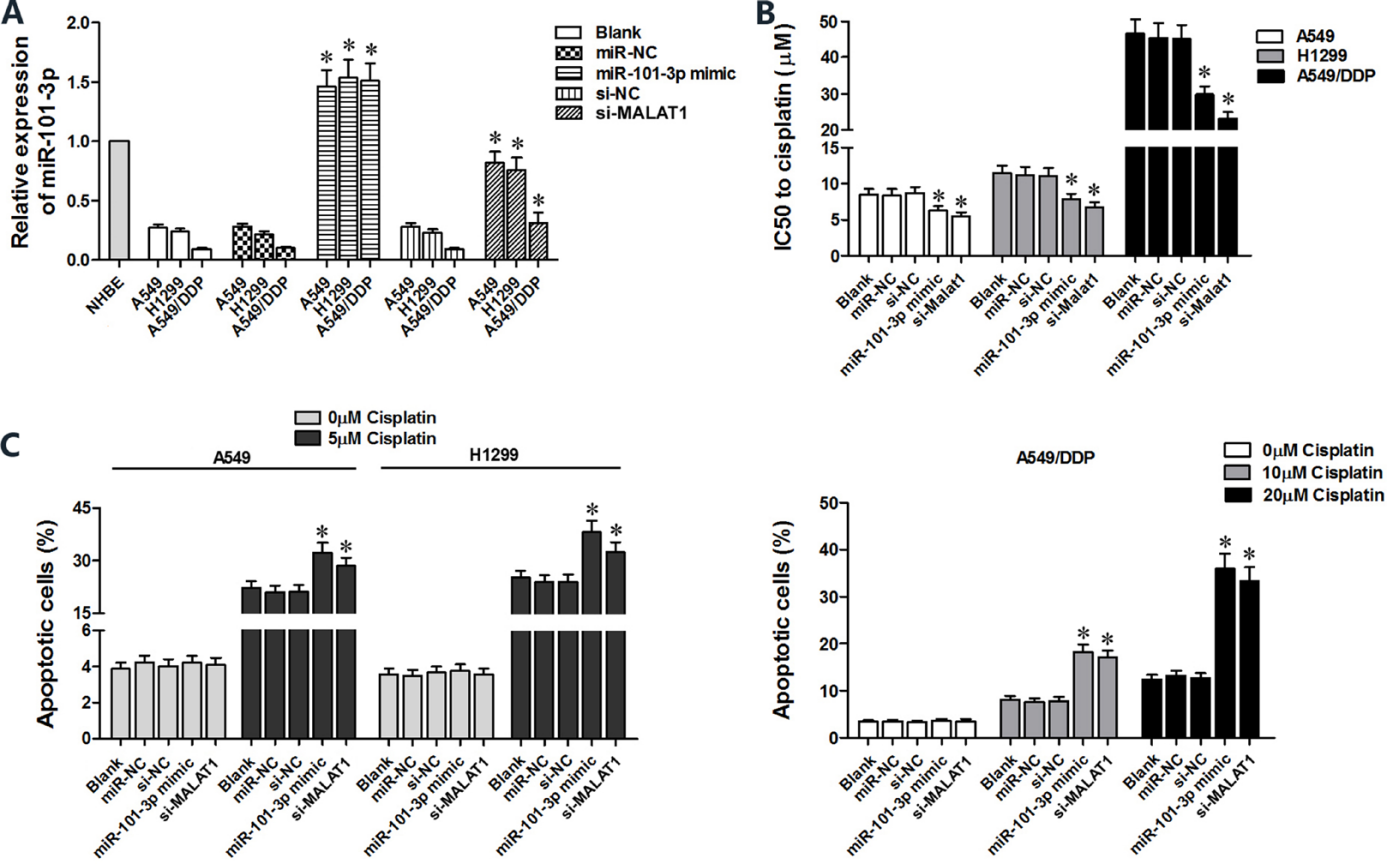
High miR-101-3p levels enhance cisplatin-induced apoptosis in lung cancer cells (**A**) QRT-PCR analysis of miR-101-3p levels in A549, H1299 and A549/DDP cells transfected with miR-101-3p mimics, si-MALAT1 or their corresponding negative controls, si-NC or miR-NC at 48 h post-transfection. (**B**) CCK-8 viability assay results show IC_50_ values of cisplatin in lung cancer cells transfected with si-MALAT1, miR-101-3p mimic or their corresponding negative controls, si-NC or miR-NC at 48 h post-transfection. (**C**) Flow cytometry analysis of apoptosis (AnnexinV^+^ PI^+^) in lung cancer cells treated with cisplatin, 48 h after transfection with si-MALAT1, miR-101-3p mimics or their corresponding negative controls, si-NC and miR-NC. Error bars represent mean ± S.D. from triplicate experiments.

We transfected miR-101-3p mimics in A549, H1299 and A549/DDP cells to determine role of miR-101-3p in cisplatin-induced apoptosis (Figure 4A). CCK8 assay showed that miR-101-3p upregulation decreased IC_50_ for cisplatin in lung cancer cells (Figure 4B). Lung cancer cells tranfected with miR-101-3p mimics or si-MALAT1 showed increased apoptosis upon cisplatin treatment than the corresponding controls (Figure 4C). MALAT1 knockdown and miR-101-3p upregulation also sensitized A549/DDP cells to cisplatin treatment (Figure 4C). These data demonstrate that miR-101-3p upregulation or MALAT1 knockdown increases cisplatin sensitivity of lung cancer cells.

### MALAT1 modulates cisplatin-sensitivity of lung cancer cells by sequestering miR-101-3p from binding to 3’-UTR of MCL1 mRNA

**Figure 5:**
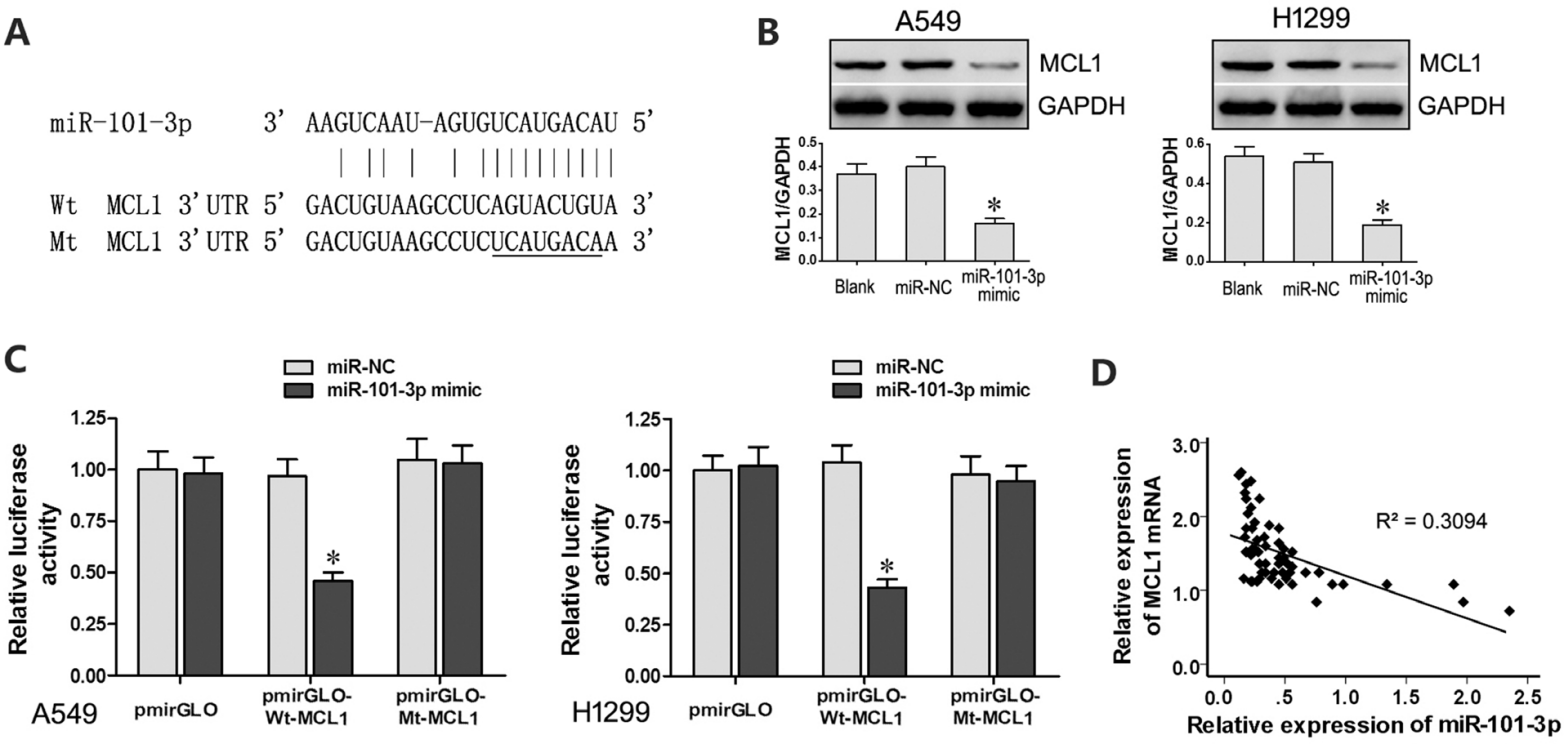
*MCL1* is a target of miR-101-3p (**A**) Predicted binding site of miR-101-3p in the 3’-UTR of MCL1 mRNA based on Targetscan analysis. (**B**) Representative western blots show MCL1 levels in A549 and H1299 cells transfected with miR-101-3p mimics or miR-NC. GADPH was used as internal control. (**C**) Relative luciferase activity is decreased in cells transfected with Wt-MCL1 (wild type miR-101-3p binding site in 3’UTR) and miR-101-3p mimics than in cells transfected with Mt-MCL1 (mutant miR-101-3p binding site in 3’UTR) and miR-101-3p mimics. This demonstrates that miR-101-3p directly binds to 3’-UTR of MCL1 mRNA. (**D**) Pearson’s analysis shows correlation between MCL1 and miR-101-3p levels in tissue samples from lung cancer patients sensitive or resistant to cisplatin (*n* =28/group; Pearson’s co-efficient [R] = - 0.556). Error bars represent mean ± S.D. from triplicate experiments.

We identified myeloid cell leukemia-1 (MCL-1) as a putative target of miR-101-3p based on TargetScan analysis (www. targetscan.org; Figure 5A). Western blot analysis showed that miR-101-3p mimics decreased MCL1 protein levels in A549 and H1299 cells than in controls (Figure 5B). Moreover, dual luciferase reporter assay showed that co-transfection of miR-101-3p mimics and Wt-MCL1 plasmid decreased relative luciferase activity than in cells co-transfected with miR-101-3p mimics and Mt-MCL1 plasmid (Figure 5C). Furthermore, qRT-PCR analysis of lung cancer tissues revealed that MCL-1 expression negatively correlates with miR-101-3p levels (R = -0.556, Figure 5D). These results demonstrated that MCL1 was a functional target of miR-101-3p.

**Figure 6:**
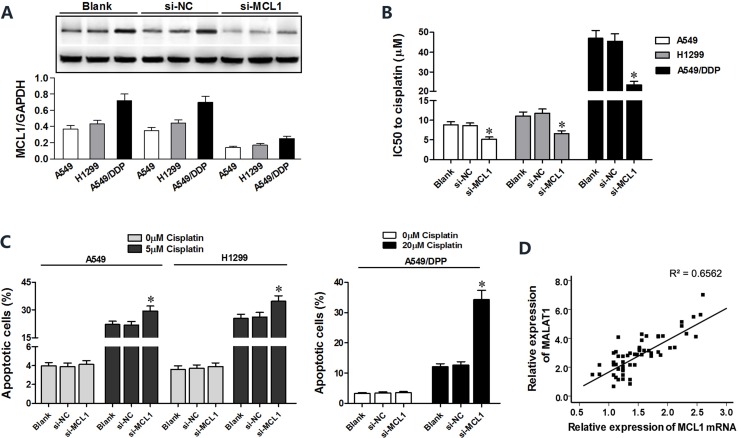
*MCL1* knockdown sensitizes lung cancer cells to cisplatin (**A**) Representative western blots show MCL1 levels in A549, H1299 and A549/DDP cells transfected with si-MCL1 or control (si-NC) at 48 h post-transfection. GADPH was used as internal control. (**B**) CCK-8 viability assay results show IC_50_ value of cisplatin in lung cancer cells transfected with si-MCL1 or si-NC at 48 h post-transfection. (**C**) Flow cytometry analysis of apoptosis in lung cancer cells transfected with si-MCL1 or si-NC and treated with cisplatin at 48 h post-transfection. (**D**) Pearson’s analysis shows correlation between MCL1 and miR-101-3p levels in tissue samples from lung cancer patients that are sensitive or resistant to cisplatin (*n* = 28/group; Pearson’s correlation co-efficient [R] = 0.810). Error bars represent as mean ± S.D. from triplicate experiments.

**Figure 7:**
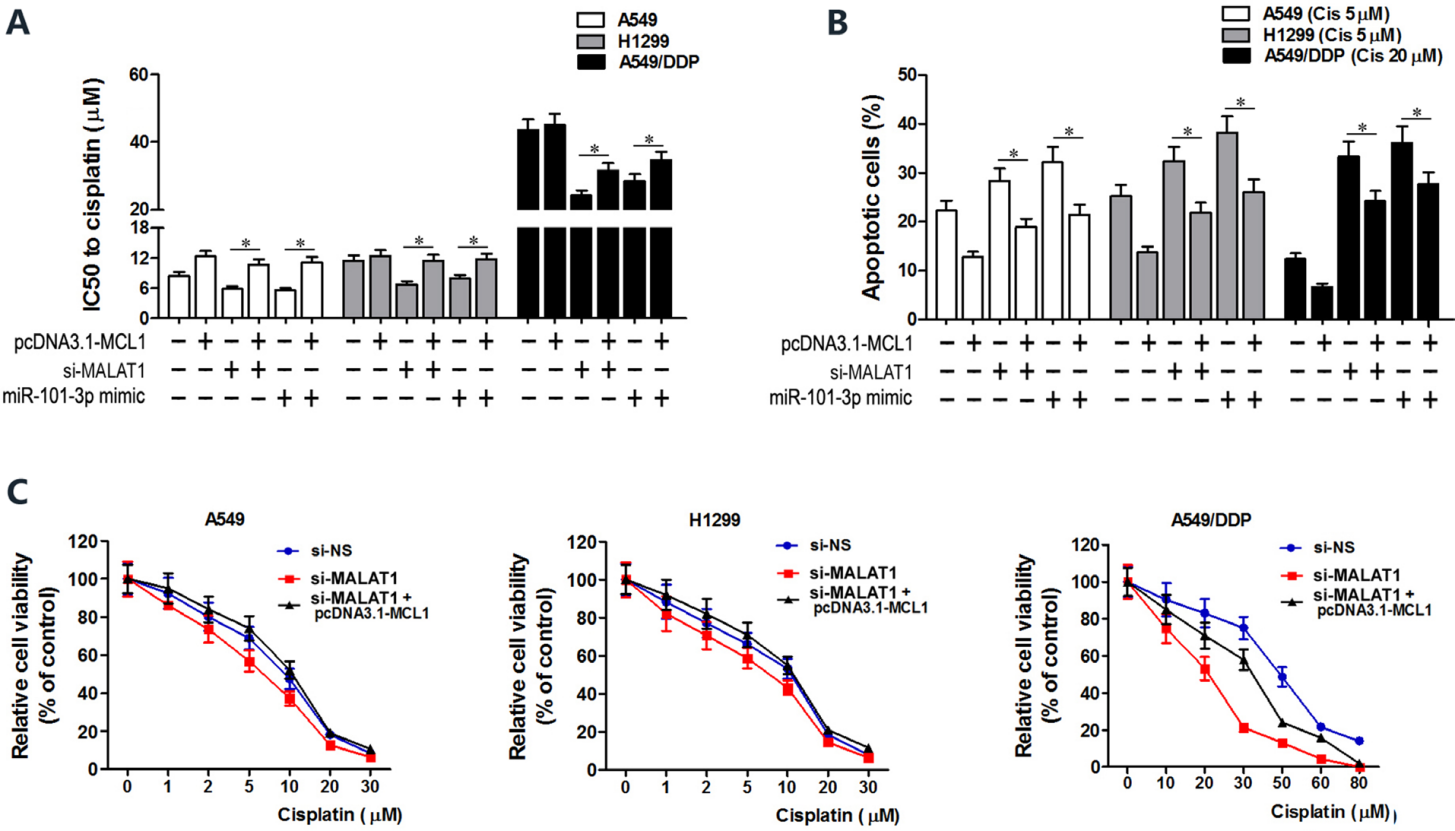
MCL1 upregulation reverses cisplatin sensitivity induced by miR-101-3p mimics and MALAT1 knockdown (**A**) CCK-8 viability assay shows IC_50_ values of cisplatin in lung cancer cells transfected with pcDNA3.1-MCL1, si-MALAT1, miR-101-3p mimics, pcDNA3.1-MCL1+siMALAT1 and pcDNA3.1-MCL1+miR-101-3p mimic at 48 h post-transfection. (**B**) Flow cytometry analysis shows cisplatin-induced apoptosis in lung cancer cells transfected with pcDNA3.1-MCL1, si-MALAT1, miR-101-3p mimics, pcDNA3.1-MCL1+siMALAT1 and pcDNA3.1-MCL1+miR-101-3p mimic at 48 h post-transfection. (**C**) Cisplatin-sensitivity in MALAT1 knockdown A549, H1299 and A549/DDP cells is reversed by overexpressing MCL1. Cisplatin-sensitivity of cells was determined with CCK8 assay. Error bars represent as mean ± S.D. from triplicate experiments.

MCL1 is a member of Bcl2 family, which is essential for survival in a variety of cell types. We performed MCL1 knockdown in A549, H1299 and A549/DDP cells to investigate its role in lung cancer (Figure 6A). Both CCK8 and flow cytometry assays showed that MCL1 knockdown sensitized lung cancer cells to cisplatin by decreasing the IC_50_ of cisplatin and increasing apoptosis (Figure 6B–6C). There was positive correlation between MALAT1 and MCL1 mRNA expression in lung cancer tissues (Figure 6D). Then, we cloned MCL1 cDNA sequence lacking the 3’-UTR into pcDNA3.1 plasmid (pcDNA3.1-MCL1) and transfected into lung cancer cells to express MCL1 that was insensitive to MALAT1 or miR-101-3p expression. Subsequently, we transfected A549, H1299 and A549/DDP cells with miR-101-3p mimics, si-MALAT1 or pcDNA3.1-MCL1 individually or in combination. CCK8 and flow cytometry analysis showed that miR-101-3p upregulation and MALAT1 knockdown increased cisplatin-induced apoptosis in lung cancer cells (Figure 7A–7C). However, upregulation of MCL1 partially reversed these effects (Figure 7A–7C). Taken together, these data suggest that miR-101-3p/MALAT1/MCL1 axis is a potential therapeutic target to increase sensitivity of cisplatin-resistant lung cancer cells.

## DISCUSSION

In this study, we demonstrated that lncRNA MALAT1 acts as a sponge to sequester miR-101-3p from its target, MCL1. We demonstrated that MALAT1 knockdown leads to miR-101-3p upregulation, which sensitizes lung cancer cells to cisplatin by downregulating MCL1 expression. MALAT1 is a potential prognostic and diagnostic biomarker in bladder cancer, lung cancer, nasopharyngeal carcinoma and osteosarcoma [19–22]. In most studies, high MALAT1 expression correlates with metastasis, invasion and chemoresistance of cancers. However, Han *et al.* showed that MALAT1 acted as a tumor suppressor by decreasing ERK/MAPK signaling in gliomas [23]. This indicates that MALAT1 function may differ with cell types and its specific molecular interactions. We showed that high MALAT1 expression in lung cancer tissues is inversely correlated with overall survival. Moreover, lung cancer patients can be distinguished as cisplatin-sensitive or cisplatin-resistant based on MALAT1 expression. MALAT1 knockdown increased cisplatin cytotoxicity of lung cancer cells by promoting apoptosis and reduced tumor growth in xenograft mouse models. These findings prompted us to investigate the role of MALAT1 in cancer chemoresistance in greater detail.

In recent times, competing endogenous RNA (ceRNA) hypothesis has come into prominence, wherein lncRNAs act as miRNA sponges by sequestering their target miRNAs and affect gene expression indirectly [24]. There is growing evidence that MALAT1 acts as a ceRNA with profound impact on tumorigenesis. Zhang *et al* demonstrated that MALAT1 promotes progression of gastric cancer via miR-202/Gli2 axis [25]. Chen *et al* showed that MALAT1 induced chemoresistance to temozolomine in glioblastoma multiforme by sequestering miR-203, which increased thymidylate synthase expression [26]. In lung cancers, several studies showed that MALAT1 regulates proliferation, metastasis and invasion of cancer cells [27, 28]. However, its role in chemoresistance of lung cancer has not been well studied. In our study, bioinformatics analysis demonstrated a putative binding site in MALAT1 for miR-101-3p. We further demonstrated direct binding between miR-101-3p and MALAT1 by dual luciferase reporter and RIP assays. MiR-101-3p is a tumor suppressor, which promotes apoptosis of endothelial cells and affects viability and invasiveness of squamous cell carcinoma [29–33]. In this study, we demonstrated high MALAT1 and low miR-101-3p levels in lung cancer A549 and H1299 cells. Interestingly, the cisplatin-resistant cell line, A549/DDP shows lower miR-101-3p and higher MALAT1 levels than the parental A549 cells. Moreover, miR-101-3p mimics and si-MALAT1 transfection enhanced cisplatin-induced apoptosis in the lung cancer cells. These results suggest an inverse relationship between miR-101-3p and MALAT1 in the lung cancer cells and their role in cisplatin sensitivity.

Our study showed that lncRNA MALAT1 elicits its biological effects by acting as a sponge for miR-101-3p, which affects the ability of miR-101-3p to bind to its targets. We demonstrated that MCL1 was a miR-101-3P target based on Targetscan analysis and dual luciferase reporter assay. Moreover, miR-101-3p mimics downregulated MCL1 expression in lung cancer cells, thereby confirming that miR-101-3p negatively regulates MCL1. Myeloid cell leukemia 1 (MCL1) is an anti-apoptotic Bcl2 family member that is associated with poor prognosis of multiple myeloma and breast cancer [34, 35]. In non-small cell lung cancer, cells with high MCL1 levels are more resistant to cisplatin. Downregulation of MCL1 increases cisplatin-induced apoptosis in lung cancer cells [36]. Moreover, MCL1 inhibitor S63845 promotes apoptosis in multiple myeloma, leukemia and lymphoma cells [37]. In our study, the cisplatin-resistant A549/DDP cells showed higher MCL1 levels than the parental A549 cells. Silencing MCL1 by miR-101-3p mimics enhanced cisplatin cytotoxicity in the lung cancer cells. Recently, researchers investigated miR101/MCL1 signaling in other cancers. He *et al.* showed that miR-101 sensitized liver cancer cells to doxorubicin by targeting MCL1 [38]. Liu *et al.* showed that miR-101increased paclitaxel sensitivity in human triple negative breast cancer cells (MDA-MB-435) by suppressing MCL1 expression [39]. These results confirm that MALAT1 regulates MCL1 expression by sequestering miR-101-3p directly.

**Figure 8:**
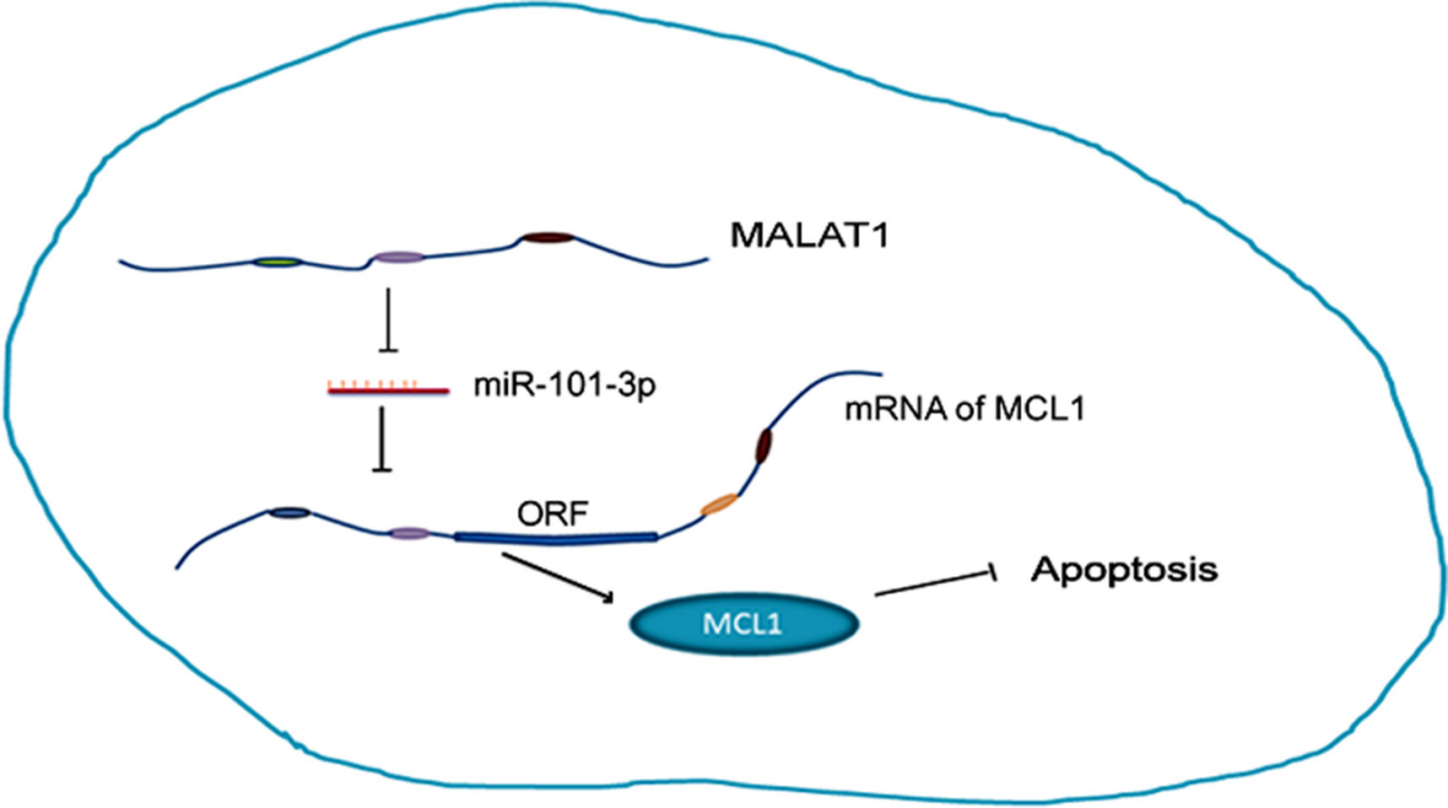
MALAT1 promotes cisplatin resistance by sequestering miR-101-3p and enhancing MCL1 expression through the MALAT1/miR-101-3p/MCL1 axis

In conclusion, we demonstrate that higher MALAT1 expression is a negative prognostic biomarker in lung cancer and indicates cisplatin resistance. MALAT1 promotes cisplatin resistance by sequestering miR-101-3p and enhancing MCL1 expression (Figure 8). Thus, our study indicates that MALAT1 is a potential therapeutic target in cisplatin-resistant lung cancer patients.

## MATERIALS AND METHODS

### Lung cancer tissues samples

Patients with confirmed lung adenocarcinoma were enrolled at The First Affiliated Hospital of Zhengzhou University from March, 2011 to July, 2013 and were classified as cisplatin-sensitive and cisplatin-resistant (*n* = 28/group). Tissue samples were obtained from each patient with CT guided biopsy, snap-frozen in liquid nitrogen and preserved at -80°C for subsequent RNA or protein extractions. Patients were enrolled into the cisplatin-resistant group based on the criteria described in a previous study [40]. This study was approved by the medical research ethics committee of Zhengzhou University. Written informed consent was obtained from all patients that participated in this study.

### Lung cancer cell lines and cell culture

Five lung adenocarcinoma cell lines (A549, H1299, H469, SPC-A1 and A549/DDP) and the normal human bronchial epithelium (NHBE) cell line were purchased from Cell Bank, Chinese Academy of Science (Shanghai, China). All cells were cultured in DMEM medium (Gibco, CA, U.S.) supplemented with 10% fetal bovine serum (Gibco, CA, U.S.), 100 U/ml penicillin and 100 μg/ml streptomycin at 37°C and 5% CO_2_. Moreover, 10μM cisplatin was added into the A549/DDP culture medium to maintain its cisplatin-resistance phenotype.

### RNA extraction and qRT-PCR

Total RNAs of all tissues and cell lines were extracted with the Trizol reagent (Invitrogen, CA, U.S.) according to the manufacturer’s instructions. Quantitative RT-PCR (qRT-PCR) was performed to estimate MALAT1, miR-101-3p and MCL1 mRNA levels with Power SYBR Green Assay (Applied Biosystems, MA, U.S.) in a 7500 Fast PCR system (Applied Biosystems, MA, U.S.) based on manufacturer’s protocol. GAPDH and U6 were used as internal controls for lncRNA and miRNA, respectively. The primers sequences used were as follows:

**Table:** 

Gene	Primer sequences (5’-3’)
MCL1 MALAT1 GAPDH U6 miR-101-3p	Forward 5′-CGGACTCAACCTCTACTGTG-3′ Reverse 5′-TTTGATGTCCAGTTTCCGAAGC-3′ Forward 5’-GCGACGAGTTGTGCTGCTATCT-3’ Reverse 5’-ACACTGCTCTGGGTCTGCTTTT-3’ Forward 5’-GAGTCAACGGATTTGGTCGT-3′ Reverse 5’-CATGGGTGGAATCATATTGGA-3′ RT-primer 5’-GTCGTATCCAGTGCAGGGTCCGAGGTATTCGCA CTGGATACGACACGATT-3’ Forward 5’-TCCGATCGTGAAGCGTTC-3’ Reverse 5’-GTGCAGGGTCCGAGGT-3’ RT-primer5’-GTCGTATCCAGTGCAGGGTCCGAGGTATTCGCACTGGATACGACATGTCAT-3’ Forward 5’-TCCGAAAGTCAATAGTGTC-3’ Reverse 5’-GTGCAGGGTCCGAGGT-3’

The relative expression of RNAs was calculated by 2^-ΔΔCt^ method. All experiments were performed in triplicate.

### Transfection protocol

The control (si-NC) and MALAT1 (si-MALAT1) shRNAs were cloned into lentiviral vectors (GenePharma Supersilencing Vector, GenePharma, Shanghai, China) and the lentiviral particles were packaged by Genepharma. A549, H1299 and A549/DDP cells were transfected for 48 h with 50 nM of miR-101-3p mimics, miR-101-3p inhibitor, si-MCL1 or its corresponding negative control siRNA (si-NC; all from Genepharma) with Lipofectamine 2000 (Invitrogen, CA, U.S.) according to the manufacturer’s protocols.

### Cell Counting Kit-8 assay

Cisplatin cytotoxicity was assessed with the Cell Counting Kit-8 (CCK-8) assays. Briefly, we prepared 0, 1, 2, 5, 10, 20, 30, 50, 60 and 80 μM cisplatin solutions in DMSO. A549 and H1299 cells were incubated with 0, 1, 2, 5, 10, 20, 30 μM cisplatin, whereas A549/DDP cells were incubated with 0, 20, 30, 50, 60, 80 μM cisplatin for 24h in 96 well plates (5 x 103 cells/well). Then, all the wells were incubated with CCK8 reagent for 4h. The absorbance of each well was read in a microplate reader at 450nm. The cells incubated in DMSO alone were set as control group (100% survival) and used as reference to determine relative cell viability at different concentrations of cisplatin. The concentration of cisplatin that accounted for 50% lethality was determined from the viability versus cisplatin concentration curves. The experiments were repeated thrice.

### Flow cytometry

Lung cancer cells (A549, H1299 and A549/DDP) were seeded in 6-well plates and treated with cisplatin for 24 h (A549 and H1299 cells were incubated with 0 and 5 μM cisplatin; A549/DDP cells were incubated with 0, 10 and 20 μM cisplatin). Then, the cells were washed with ice-cold PBS twice and stained with Annexin V-FITC/propidium iodide (Invitrogen, CA, U.S.) in the dark for 15min according to manufacturer’s instructions. Then, the percentage of apoptotic cells in each sample was determined in a BD FACSCalibur flow cytometer (BD Biosciences, Franklin Lakes, NJ, U.S.). The experiments were repeated thrice.

### Xenograft mouse model

BALB/c nude mice (female, 4–6 weeks old) were purchased from Beijing Vital River Laboratory Animal Technology center (Beijing, China) and kept in a temperature-controlled, sterile environment with 12 h light and dark cycles. Animals were randomly distributed into four groups (*n* = 5/group) that were subcutaneously injected under the left forelegs with siMALAT1- or siNC-transfected A549 and H1299 cells (5×10^6^ in 0.2 ml DMEM medium without serum). When the tumors were detected using an *in vivo* small animal imaging instrument (IVIS Spectrum *in vivo* Imaging System, PerkinElmer, Hopkinton, Massachusetts, U.S.), the mice were treated with 2 mg/Kg cisplatin via intraperitoneal injection for 5 days. The growth of xenograft tumors was determined every week using bioluminescence imaging for four weeks. Then, the mice were euthanized and the tumors were excised and weighed.

### Dual luciferase reporter assays

We performed dual luciferase reporter assays to verify the direct interactions between MALAT1 and miR-101-3p as well as miR-101-3p and MCL-1 mRNA. We used PCR to amplify MALAT1 cDNA containing predicted miR-101-3p binding site and the 3’UTR of *MCL-1* from human genomic DNA and cloned the PCR products downstream of the luciferase gene in the pmirGLO vector. Then, we co-transfected pmirGLO–Wt-MALAT1 or –Mt-MALAT1 with miR-101-3p mimics or scrambled miRNA (miR-NC) into 70–80% confluent A549 and H1299 cells with Lipofectamine 2000 (Invitrogen, CA, U.S.) according to the manufacturer’s protocols. Moreover, pmirGLO–Wt-MCL1 and pmirGLO–Mt-MCL1 were transfected into A549 and H1299 cells similarly. pRL-TK plasmid was transfected as an internal control. The luciferase activity was determined with a luciferase assay kit (Promega, WI, U.S.) at 48 h after transfection.

### RNA immunoprecipitation (RIP) assay

RNA immunoprecipitation (RIP) assay was performed using an EZ-Magna RiP Kit (Millipore, Billerica, MA, USA) in accordance with the manufacturer’s instructions. A549 and H1299 were lysed at 70–80% confluence in RIP lysis buffer, and then incubated with magnetic beads conjugated with human anti-Ago2 antibody (Millipore) and normal mouse IgG control (Millipore) in RIP buffer. The RNAs in the immunoprecipitates were isolated with Trizol reagent and analyzed by qRT-PCR.

### Western blotting

Total protein lysates were prepared from different groups of cells with RIPA lysis buffer (Solarbio, Beijing, China) containing proteinase inhibitor cocktail (Thermo Scientific) and quantified with a BCA kit (Solarbio, Beijing, China). Equal amounts of total protein were loaded onto 10% SDS-PAGE gel and electrophoresed at 50 mV for 4 h followed by transferring onto PVDF membrane (Invitrogen, CA, U.S.) at 80 mV. The membranes were then blocked with 5% non-fat milk for 2 h at room temperature. Subsequently, the membrane was incubated with rabbit anti-human MCL1 polyclonal antibody (sc-819, Santa Cruz, CA, U.S.) at 4°C overnight. Then, the blots were washed four times with PBST and incubated with HRP-conjugated anti-rabbit secondary antibody (Santa Cruz, CA, U.S.) for 2 h at room temperature. Then, the blots were visualized with the FluorChem imaging system (ProteinSimple, CA, U.S.) and quantified with the IPP image analysis software. GADPH (Beijing Dingguo Changsheng Biotechnology Co., Ltd, Beijing, China) was used as internal control. All experiments were performed in triplicate.

### Statistical analysis

All statistical analyses were performed with the SPSS 21 software (SPSS Inc., IBM, Armonk, New York, U.S.). Data were presented as mean ± S.D. from three independent experiments. One-way ANOVA was used to compare multiple groups, whereas, a two-tailed unpaired Student’s *t*-test was used to analyze differences between two groups. Kaplan Meier survival curves were used to determine overall survival based on log rank test. Pearson correlation analysis was used to evaluate the relationship between two groups. *P* < 0.05 was considered statistically significant.

## Abbreviations

MALAT1: Metastasis-associated lung adenocarcinoma transcript 1
lncRNA: Long noncoding RNA
FAL1: Focally Amplified Long Noncoding on Chromosome 1
HOTAIR: HOX transcript antisense RNA
DDP: Cis-diamminodichloroplatinum
RISC: RNA-induced silencing complex
RIP assay: RNA immune-precipitation assay
CCK8: Cell Counting Kit-8
miRNA: microRNA
MCL1: Myeloid cell leukemia 1
S.D: standard deviation
